# Implementation of RedCap data systems in Kenyan psychiatric rehabilitation: a community case study

**DOI:** 10.3389/fpsyt.2024.1386249

**Published:** 2025-01-14

**Authors:** Neal Patel, Matthew Turissini, Florence Jaguga, Julius Barasa, Lily Okeyo, Mercy Kimaiyo, Gilliane Kosgei, Naomi Kipkorir, Edith Kamaru Kwobah

**Affiliations:** ^1^ School of Medicine, Indiana University, Indianapolis, IN, United States; ^2^ Department of Medicine, School of Medicine, Indiana University, Indianapolis, IN, United States; ^3^ Department of Mental Health, Moi Teaching and Referral Hospital, Eldoret, Kenya; ^4^ Academic Model Providing Access to Healthcare (AMPATH) Mental Health Care Program, Eldoret, Kenya

**Keywords:** LMIC, Kenya, community case study, REDCap, psychiatric rehabilitation

## Abstract

Data collection systems are vital in implementation of interventions within patient care as they allow us to better understand and improve the care given. This is equally true in the field of psychiatric rehabilitation, which has been steadily growing in the medical world for the past 20 years. It has recently been a focus of psychiatric medicine in Kenya with the Kenya Mental Health Action Plan 2021-2025 calling for improvement of care in the field. With psychiatric rehabilitation growing in low- and middle-income countries in recent years studies have come out showing the effects of newly employed programs and stressing the need for data collection practices that can be used to encourage best practices in local communities. This paper describes the implementation of data systems for the Moi Teaching and Referral Hospital Nawiri Skills and Recovery Centre (Nawiri) in Eldoret, Kenya. The goals of the data systems were 1) To collect and organize information for continuous quality improvement of services at Nawiri, 2) To collect information for reporting to MTRH leadership, Ministry of Health as well as private funders, 3) To provide data to assess outcomes of people who receive services at Nawiri to create evidence for effective interventions to help create policy to make psychiatric rehabilitation more available to people living with Severe mental illness in Kenya. Lessons learned from data systems implementation included insights into ease of data collection, adaptability of data instruments to fit facility needs, and ability to reproduce RedCap data collection in other settings.

## Introduction

Data collection systems are vital to the functioning of care programs as they allow for better understanding of the effects of interventions. Proper data collection, which encompasses the selection, collection, storage, and quality improvement, can lead to more efficient changes and better care for patients. This is especially true for projects that involve qualitative measures of well-being in addition to quantitative measures. A data collection tool that is commonly employed in research and other endeavors is RedCap. This system allows for the maintenance of multiple patient data forms in a secure database. The flexibility of this system and wide range of capabilities in the collection of qualitative data make it a valuable service (1).

There are many data collection systems used by studies and projects. Historically, pen and paper data capture methods have been used in many resource-limited settings due to “being financially unfeasible or difficult to support technically.” (2) However, electronic data capture has advantages such as lower error rate and lower cost per entered question compared to pen and paper counterparts (2). Advances in the field such as the evolution of RedCap into a low cost, easy to use tool have also opened the door for electronic data collection uses. Advantages to RedCap specifically include an easy-to-use designer, ability to collaborate with multiple institutions in creation, and low maintenance required after initial setup. In addition, there is no limit to the number of users that can be added as collaborators on a RedCap study and no limit to the number of records that can be stored in a database. There is also a built-in system for data access to allow for tiered access to the database for data collectors, managers, and administrators. Licensing for RedCap is free to non-profit institutions and involves the signing of an end user licensing agreement to obtain access. After an agreement is signed by an institution, installation and maintenance are performed by the individual institution and their IT department. Limitations do include the lack of large dataset standards that can be used to compare studies within the software (1). Overall, the data system has appeal in a low resource setting and the ease of use lends itself to projects that aim to venture into new areas within a field such as psychiatry, namely psychiatric rehabilitation.

Psychiatry is a field that has shifted in dynamic and scope in the past couple of decades. Care has shifted away from psychiatric institutionalization to psychiatric beds in general hospitals (3). There has also been an increase in efforts to develop mental health in lower- and middle-income countries. The 2015 United Nations Sustainable Development Goals set mental health goals for the first time ever (4). Kenya has experienced several advancements in mental health over the past decade including strengthening of governance (5) and an increase in the number of mental health care providers (6). These changes help increase the availability for inpatient and outpatient mental health care in Kenya, but no psychiatric rehabilitation programs existed in the public sector in Kenya until recently. Psychiatric rehabilitation is defined as “to help individuals with persistent and serious mental illness to develop the emotional, social and intellectual skills needed to live, learn and work in the community with the least amount of professional support” (7).

Mental health rehabilitation has been studied for the past couple of decades in Europe and the US, though more so recently. A study conducted in the UK in the early 2000’s examined the definition of “mental health rehabilitation” by asking UK providers their thoughts and coming up with a collated definition: ‘A whole systems approach to recovery from mental illness that maximizes an individual’s quality of life and social inclusion by encouraging their skills, promoting independence and autonomy in order to give them hope for the future and which leads to successful community living through appropriate support’ (Killaspy et al., 2005) (8). One of the first large studies to examine the effects of mental health rehabilitation in community settings outside of hospitals was the TAPS (Team for the Assessment of Psychiatric Services) project which examined a cohort of patients who were released from long-stay mental health institutions to assisted community living. The study found that transitionary community homes resulted in less crime and homelessness as well as greater patient satisfaction. However, another finding of the study was that more research needs to be done to truly determine the benefit vs cost of community centers (9). This sentiment of needing more research and better standardization has been echoed in many studies since including one systematic review of over 100 articles which found it difficult to compare different studies due to differences in terminology and service characteristics of different studies (10).

Lack of robust mental health research is a significant issue in lower- and middle- income countries where mental health is a growing field. Little research has been done on mental health rehabilitation and there are few psychiatric rehabilitation centers to begin with. There is evidence in lower- and middle- income countries of benefit from client-centered vocational and other rehabilitation interventions for patients with severe mental illness (11). A study conducted in South Africa showed that provider side biases were reduced, and confidence and knowledge increased in community care workers with training in mental health services (12). A different group in Ethiopia discovered the plethora of community resources that can be available even in rural settings of Africa, using mapping techniques to identify community resources that could integrate in Ethiopian mental health care (13).

Despite the benefits shown by preliminary research, implementation of increased community-based mental health services is lagging behind. A study done in 2011 looked at the mental health policy in four countries: Ghana, South Africa, Uganda, and Zambia. The study found that all four of the countries encouraged further growth and development of mental health resources but failed to outline any method or plan to tackle the problems. None of the countries specified sources of funding for any projects and none of the countries spoke of de-institutionalization and the need for mental health rehabilitation (14).

In the years since 2011, however, there have been advances and insights into supplemental mental health programs. A study in South Africa in 2019 showed that attendance at a daily occupational therapy metal health center significantly reduced inpatient hospital days (15). A 2021 study in Nigeria showed the benefit of a dedicated mental health rehabilitation center by polling individuals with schizophrenia who followed up with outpatient full-service mental health centers. The researchers found that rehabilitation programs that were involved with hospitals and also allowed individuals increased choice and capacity in vocational training led to better satisfaction and perception. The study, like many others, also recommended increased uniformity in care based on best practices (16).

In Kenya, there has been an increased focus on psychiatric rehabilitation in recent years, with the Kenya Mental Health Action Plan 2021-2025 calling for improved care in this field (17). With the novelty of this intervention in the Kenyan population, no best practices have been identified and no best practices were listed in the Action Plan as well.

As individuals participate in mental health rehabilitation and psychosocial interventions, there is a need to develop data systems to track changes and outcomes. The data collections methods must fit within the cultural and social dynamics present with Kenyan patients to ensure proper understanding and interpretation of questions. Psychiatric rehabilitation can be similar to both inpatient and outpatient clinical settings as it focuses on intensive interventions and requires longer term follow-up of patients. Creating a data system that can cover. This allows for more accurate data collection and for results to better reflect the perspective of the patients, to assess outcomes, to improve interventions, and to affect healthcare policy related to mental health.

## Context

This paper examines the creation of data systems for the MTRH Nawiri Recovery and Skills Centre (Nawiri) in Eldoret, Kenya as well as lessons taken throughout implementation. Nawiri is the first psychiatric rehabilitation facility in the public sector in Kenya offering a 2-3 month care curriculum to help patients living with severe mental illness live more sustainably in the community. Nawiri is located in a national referral hospital and has sixteen residential spots for individuals living with severe mental illness and offers day services. The center provides therapeutic interventions including group therapy based on illness management and recovery, individual therapy, and family therapy; life skills and vocational skills training; and re-integration into their family and the community. These interventions are run by a multidisciplinary team. Clients at Nawiri set their own goals for recovery for future independence and re-integration into communities. As the Nawiri center is the first of its kind in Kenya, a secondary goal is to establish a framework and methodology that can be used to develop similar centers throughout the country.

## Key programmatic elements

One of the key elements that was instituted in Nawiri at the start was an emphasis on clear and validated data collection to assess effectiveness. We set data goals through a collaborative process with Nawiri staff to guide creation of data systems for tracking. These goals for creating the data system were:

1) To collect and organize information for continuous quality improvement of services at Nawiri.
2) To collect information for reporting to MTRH leadership, Ministry of Health as well as to partner organizations and funders.
3) To provide data to assess outcomes of people who receive services at Nawiri to create evidence for effective interventions to help create policy to make psychiatric rehabilitation more available to people living with Severe mental illness in Kenya.

To facilitate these goals, our team spent significant time deciding which metrics to collect and the best method to collect them. We emphasized time for data collection and entry in the care program to ensure staff could focus on patient care and to highlight sustainability as a consideration.

### Electronic data system selection

Given the resource-limited nature of mental health care in Kenya, we sought a data collection system that had demonstrated feasibility in similar contexts. The RedCap electronic data collection system had previously been shown to be effective and efficient even in a similar resource-limited setting in Colombia (18). There have also been many programs using RedCap in African settings including projects in Nigeria (19), South Africa (20), and Kenya (21–23). As this system also had capacity for longitudinal data collection, it was chosen for the Nawiri program. We ultimately decided to do a hybrid model of paper charts which were the norm in the setting to collect detailed care information which can often be very contextual for our patient population, with pre-chosen discrete fields and metrics entered in the RedCap system to guide the above data goals. The RedCap system that was used in this project was house by the Academic Model Providing Access to Healthcare (AMPATH) servers. AMPATH is an organization that all collaborators were a part of and includes Moi Teaching and Referral Hospital as well as multiple US academic centers as partners.

### Confidentiality

We established our data system as secure to ensure confidentiality of patient records and worked to ensure continued security over time. Paper records are kept in locked cabinets at Nawiri. REDCap is a server side database which is compliant with a number of international confidentiality standards. We ensured only necessary access to the REDCap database by determining access to the REDCap database based on role at Nawiri (Role-based Access Control). We created individual REDCap accounts for team members who did database programming, data entry, and analysis as well as Nawiri leadership. We had no staff leave during implementation to date but can remove accounts form the REDCap database server with assistance of AMPATH IT staff to remove access if people no longer need it for their jobs.

### Data value selection

We collected demographic data to aid the provision of care to clients and for the above data systems goals. We included common values such as age, sex, occupation, and education level. Added values included previous mental health diagnoses, previous mental health hospitalizations, and average expenditure per month. The added values helped better characterize the previous psychiatric history of residents at Nawiri and provide context for future changes. The data values collected to track progress were chosen to provide an objective view of patient status. The scales initially chosen for patient evaluation were the Sheehan Disability Scale and the Recovery Assessment Scale- Domains and Stages (RAS-DS) Scale. The Sheehan Disability Scale was chosen as a measure of disability in individuals with mental illness. Initially, the RAS-DS Scale was used and was chosen due to the belief that they allowed for subjective and patient-provided measures of recovery in their mental illness and re-engagement in community. The RAS-DS scale was not used long term for data collection due to difficulties with obtaining accurate data when administered. The scale also had a large number of questions which was not conducive to timing of data collection and entry as well as patient attention and engagement during administration.

Other data was instead implemented to assess the recovery and independence of the patients. The data tools used incorporated the existing forms that were used to collect patient demographics and diagnoses.

### Data collection time points

As the recovery process for mental health rehabilitation is a long one with multiple steps in reintegration into community and vocational training, longitudinal data was desirable. The data collection time points chosen for the RedCap database were Intake, Discharge, and Follow up post-exit at 2 weeks, 1 month, 3 months, 6 months, and 12 months. The frequent follow-ups after discharge were to promote recovery as part of the intervention, and to recognize any challenges in the community early after discharge, so they could be addressed to promote recovery. 6- and 12-month follow-ups were done because the ultimate goal of Nawiri was to facilitate recovery and independence in the community over longer periods.

### Data collection progress

So far, we have collected data points for 101 patients over the course of the past 2 years. We have been able to keep track of patient data from intake to the last follow-up post exit from the facility. The data has been initially collected on paper forms and then transferred to the RedCap database either the same day or within one week of collection. Data transfer has been done by Nawiri staff who have access to data entering and editing, but not editing of the forms themselves. We did not initially create a written protocol for training, but procedures were verbally put in place for data collection as highlighted above. As a majority of data entry was done by less than five total workers it was possible to orient the whole team without need of written protocols. To prevent errors in data entry RedCap does have built in data tools and data validation which prevents errors such as entering incorrect dates and missing data points. Otherwise, data entry errors were monitored by double checking and confirming with paper forms initially. Also, quarterly data monitoring was performed as we would compile quarterly reports. Eventually, the plan will be for complete data entry directly into RedCap to remove this room for error. The data is managed by institutional data managers from AMPATH. Since implementation, we have modified the RedCap database through collaboration with on-ground facility personnel to provide better ways to track insights into patient condition.

## Discussion

This community case study examines the implementation of an electronic data record system for the Nawiri Recovery and Skills Centre, a psychiatric rehabilitation facility in Eldoret, Kenya. The implementation of electronic data systems in Nawiri provided lessons and positive takeaways for data collection in psychiatric rehabilitation care in a government national referral hospital in Kenya. The main purpose of this community case study was to describe key lessons and takeaways from implementation in Nawiri that could be useful to similar projects. The key takeaways from implementation fall into the categories of ease of data collection, adaptability of the system to meet facility needs, and ease of reproduction.

Data collection in a care program is important to guide patient care, assess outcomes, and guide quality improvement efforts. However, it is important to optimize data collection systems to maximize this data collection while not making the data systems impinge on effective care system delivery – especially in resource constrained settings. Uncomplicated data collection became an issue for the RAS-DS survey after initial data instrument implementation. The survey was initially included due to the belief that it would provide reliable results and prove easy to use for administrators and patients alike. In practice, the survey was prohibitively lengthy for a care program with limited staff. The amount of staff time was an issue and clients often lost concentration or interest during the survey. There were multiple instances of patients providing the same number answer to every question in the study though we were unable to discern if this was due to administrator or patient factors. These factors, in addition to the survey itself not having been validated in a Kenyan population prior, led to the discarding of this measure for future collection and analysis. The Sheehan Disability Scale and other measures used were able to be consistently collected. These forms were implemented for intake and follow up times and allowed for examination of longitudinal trends.

We ultimately implemented a data system using a mixture of paper records and the Redcap database. There was significant benefit to the care program using long form prose descriptions in both social work and psychology documentation. Given the complexity of illness and social factors affecting many patients’ reintegration into the community, detailed descriptions of these dynamics were important for the multi-disciplinary care team to document to assist individuals through recovery. Staff felt much more comfortable and efficient doing this documentation on paper charts. So, paper charts were used for initial data entry with selected metrics then placed in a Redcap. This hybrid data system worked well at Nawiri to balance patient care, track patient outcomes, and drive quality improvement projects while also facilitating limited staff to focus the majority of their time on patient care. A fully integrated digital system is a long-term goal to consolidate data and prevent any need for data transfer. The current hybrid system allows time for the Nawiri program to ensure that the integration of the long-term data system meets all data needs of the program and is efficient and non-cumbersome for limited multidisciplinary care program staff to use. We used the Redcap data base for this project, but other database software or an electronic medical record system could have been used as long as it provided appropriate data security for patient information and a program had the capacity to program forms to track care information for the unique needs of a psychiatric rehabilitation program. Our team felt that REDCap met our needs for Nawiri and do not have any recommendations for changing REDCap out of our experience using it.

Adaptability of the newly implemented system was vital to success as data collection needed to match the sustainability model implemented at Nawiri. Through the first two years of implementation the RedCap system was changed multiple times to add measures of patient outcomes that were not initially anticipated. Measures like housing, food security, average monthly expenditure, and pre-admission and current medication status were some of these added fields which were not on the initial template. Additionally, the exit interview was amended to include more fields on discharge demographics. The addition of this outcome tracking has allowed us to better understand our patient population and collect pertinent information that can influence programming and care. Nawiri has changed in a few ways based on improved monitoring of outcome data including changing the schedule of follow-up encounters after discharge from Nawiri, adjusting tools used such as eliminating use of RAS-DS described above, and recognizing patients needing further community support or referrals. Continued use of this information will also help measure change in need for psychiatric hospitalizations after rehabilitation services, access to outpatient mental health care, and independence in the community for patients after discharge from Nawiri.

The implementation of the data system in Nawiri has promise for reproduction in other centers. The simplicity of the scales used lends itself to quicker training for administrators, though it was found that after initial training, comfort with the system and becoming efficient in data entry took more time. In the Nawiri population, the Sheehan Disability Scale and other data tools provided effective glimpses into the condition of the patients and both scales were able to integrate with existing data collection methods that combined paper and electronic means. The RedCap database itself was also conducive to adaptability as the center unearthed more data points that needed to be collected. The hybrid paper and electronic database worked well in our context to fit the prose clinical data of complicated cases and the discrete fields and would be worth considering in other resource limited settings as longer term fully electronic systems are developed. Further research is needed into data tools to assess recovery which can be validated in the Kenyan context, and which are brief enough to be used in a care program setting. The next steps for the Nawiri data collection system involve finding ways to work toward a digital system to reduce work for employees as well as to streamline current data forms for ease of use.

## Constraints

We encountered constraints related to implementation related to choice of data platform and due to implementing a novel model of care as Kenya’s first psychiatric rehabilitation center. The main constraints related to choosing RedCap as a data platform which could limit replication in other programs included licensing and infrastructure including servers and consistent internet access. Programmatic implementation of the data system was limited by no tools measuring psychiatric recovery having been validated in Kenya, staff needing to be knowledgeable or trained on RedCap programming, balancing staff time between data entry and providing care to Nawiri clients, and a need to change data metrics tracked as we identified barriers and facilitators to recovery for people living with severe mental illness in a novel care program.

## Data Availability

The original contributions presented in the study are included in the article/supplementary material. Further inquiries can be directed to the corresponding author.
